# Sphingolipids metabolism in the salivary glands of rats with obesity and streptozotocin induced diabetes

**DOI:** 10.1002/jcp.25939

**Published:** 2017-04-27

**Authors:** Marta Garbowska, Bartłomiej Łukaszuk, Agnieszka Mikłosz, Igor Wróblewski, Krzysztof Kurek, Lucyna Ostrowska, Adrian Chabowski, Małgorzata Żendzian‐Piotrowska, Anna Zalewska

**Affiliations:** ^1^ Department of Hygiene, Epidemiology and Ergonomics Medical University of Bialystok Bialystok Poland; ^2^ Department of Physiology Medical University of Bialystok Bialystok Poland; ^3^ Department of Gastroenterology and Internal Medicine Medical University of Bialystok Bialystok Poland; ^4^ Department of Conservative Dentistry Medical University of Bialystok Bialystok Poland

**Keywords:** ceramide, insulin resistance, salivary glands, sphingolipids, type 1 diabetes

## Abstract

Diabetes is considered a major public health problem affecting millions of individuals worldwide. Remarkably, scientific reports regarding salivary glands sphingolipid metabolism in diabetes are virtually non‐existent. This is odd given the well‐established link between the both in other tissues (e.g., skeletal muscles, liver) and the key role of these glands in oral health preservation. The aim of this paper is to examine sphingolipids metabolism in the salivary glands in (pre)diabetes (evoked by high fat diet feeding or streptozotocin). Wistar rats were allocated into three groups: control, HFD‐, or STZ‐diabetes. The content of major sphingolipid classes in the parotid (PSG) and submandibular (SMSG) glands was assessed via chromatography. Additionally, Western blot analyses were employed for the evaluation of key sphingolipid signaling pathway enzyme levels. No changes in ceramide content in the PSG were found, whereas an increase in ceramide concentration for SMSG of the STZ group was observed. This was accompanied by an elevation in SPT1 level. Probably also sphingomyelin hydrolysis was increased in the SMSG of the STZ‐diabetic rats, since we observed a significant drop in the amount of SM. PSG and SMSG respond differently to (pre)diabetes, with clearer pattern presented by the later gland. An activation of sphingomyelin signaling pathway was observed in the course of STZ‐diabetes, that is, metabolic condition with rapid onset/progression. Whereas, chronic HFD lead to an inhibition of sphingomyelin signaling pathway in the salivary glands (manifested in an inhibition of ceramide de novo synthesis and accumulation of S1P).

## 1 INTRODUCTION

Nowadays, diabetes mellitus is considered to be one of the major public health problems affecting hundreds of millions of people worldwide and causing serious depreciation in both life comfort and expectancy (Organization, 2016 ). Moreover, this complicated multi‐facial disorder manifests itself also by a number of oral cavity maladies (e.g., periodontal disease, burning mouth syndrome, or mucosa ulcerations) that substantially impact dental care (Leite, Marlow, & Fernandes, 2013 ; Negrato & Tarzia, 2010 ). Carda, Mosquera‐Lloreda, Salom, Gomez de Ferraris, and Peydro (2006 ) for instance, found that even up to 100% of diabetic patients may present periodontal disease in comparison with the 50% occurrence rate found in the control group. Not surprisingly, since the salivary glands are the vital organs with respect to maintaining oral health and hygiene many studies have examined the composition of the salivary glands and saliva itself (Negrato & Tarzia, 2010 ). So far, the conducted analysis have encompassed amongst others: carbohydrate metabolism (Nicolau, de Matos, de Souza, Neves, & Lopes, 2005 ; Nogueira, Santos, & Nicolau, 2005 ), protein levels (Stewart et al., 2016 ), electrolytes (Lasisi & Fasanmade, 2012 ), oxidative damages (Knas, Maciejczyk, Daniszewska, et al., 2016 ; Knas, Maciejczyk, Sawicka, et al., 2016 ), or the presence of immunoglobulin (Metidieri et al., 2012 ; Malicka, Kaczmarek, & Skoskiewicz‐Malinowska, 2015 ). Remarkably, aside from our recent papers on this topic (Matczuk, Zalewska, Lukaszuk, Garbowska, et al., 2016 ; Matczuk, Zalewska, Lukaszuk, Knas, et al., 2016 ) scientific reports regarding salivary glands lipid composition are virtually non‐existent. This seems to be quite odd given the well‐established causative link between lipid over‐accumulation (e.g., diacylglycerols, triacylglycerols, and ceramides) and impaired tissue/organ functioning found in skeletal muscles (Kurek et al., 2015 ; Lukaszuk, Miklosz, Chabowski, & Gorski, 2015 ), liver (Chabowski et al., 2013 ; Konstantynowicz‐Nowicka, Harasim, Baranowski, & Chabowski, 2015 ), heart (Harasim et al., 2015 ), and adipose tissue (Matravadia, Zabielski, Chabowski, Mutch, & Holloway, 2016 ). Based on the above‐information one may postulate a significant role played by lipids in the acquisition of the salivary glands diabetic phenotype. Therefore, in the present study we seek to determine the influence of diabetes on the sphingolipid metabolism in both the parotid (PSG) and submandibular (SMSG) salivary glands.

In one of our recently published studies, we demonstrated that obesity and diet induced insulin resistance affect lipid profile in the salivary glands of rats (Matczuk, Zalewska, Lukaszuk, Knas, et al., 2016 ). In the aforementioned study, we found that the high caloric diet regime (∼60% of energy derived from fats) resulted in a reduction of membrane phospholipids concentration signifying atrophy and malfunctions of the saliva secreting organs. On the other hand, the observed increased accumulation of triacylglycerol could well be an important clinical manifestation of metabolic syndrome and type 2 diabetes in these organs, as is the case of other tissues (e.g., skeletal muscles) (Lukaszuk et al., 2015 ). Interestingly, the abovementioned disturbances in lipid metabolism were observed mainly in the glycolytic submandibular salivary glands (Matczuk, Zalewska, Lukaszuk, Knas, et al., 2016 ). Furthermore, in another study of ours (Matczuk, Zalewska, Lukaszuk, Garbowska, et al., 2016 ) we determined lipid profile of the salivary glands of rats with streptozotocin induced diabetes. In this study, we have shown that streptozotocin induced diabetes leads to a decrement in phospholipids content which indicates subsequent atrophy and malfunction in both the parotid and salivary glands. Another interesting finding of the above‐cited study was the reported increase in triacylglycerol accumulation with respect to both the parotid and salivary glands of diabetic animals (Matczuk, Zalewska, Lukaszuk, Garbowska, et al., 2016 ). The aforementioned data strongly indicate that the parotid and submandibular salivary glands are characterized by different metabolic activities. Moreover, streptozotocin induced diabetes (an experimental model mimicking clinical type 1 diabetes) exerted slightly different effect(s) on salivary glands lipid metabolism in comparison with the type 2 prediabetic state induced by high fat diet feeding (Matczuk, Zalewska, Lukaszuk, Garbowska, et al., 2016 ; Matczuk, Zalewska, Lukaszuk, Knas, et al., 2016 ).

**Figure 1:**
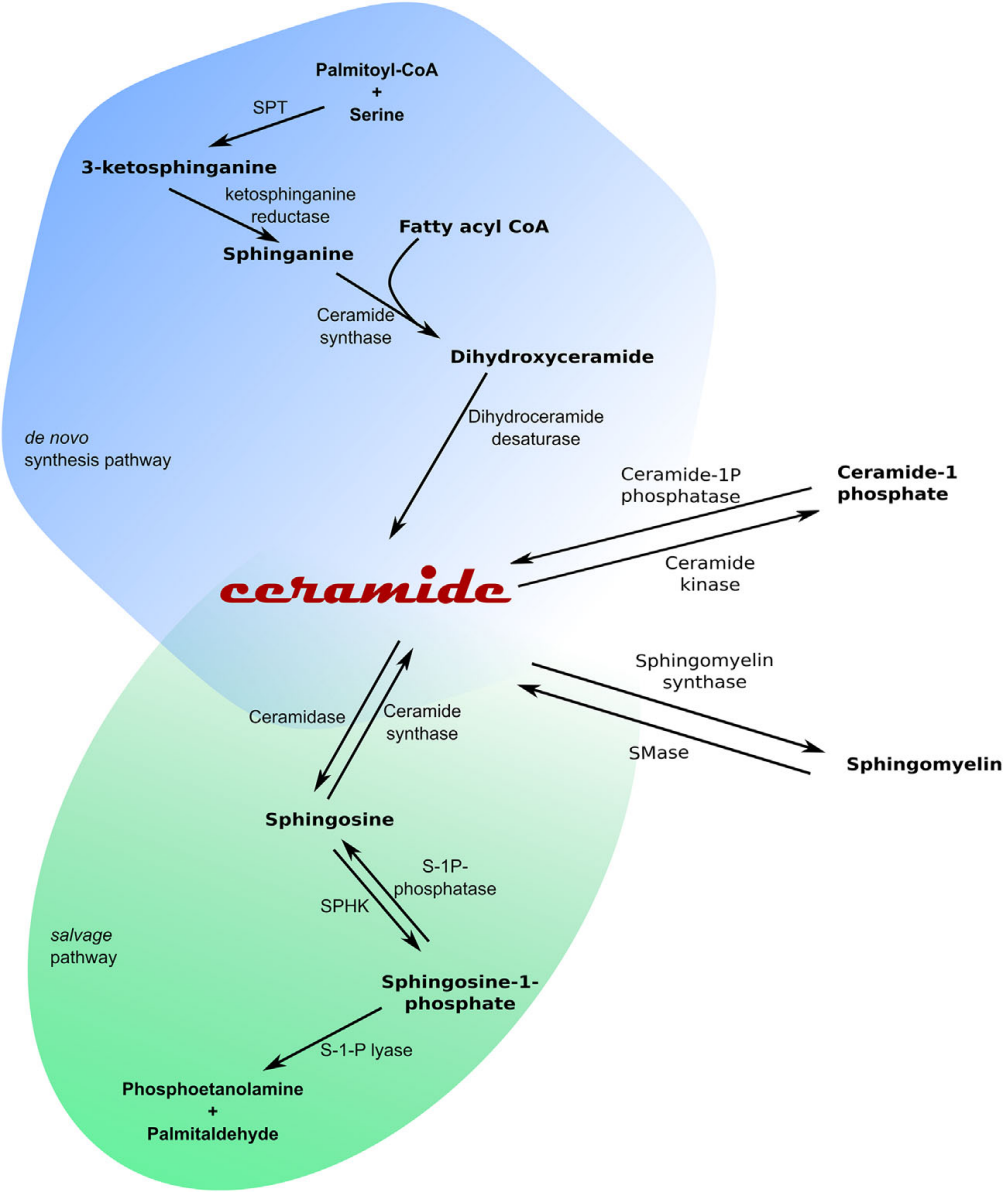
General representation of the sphingomyelin signaling pathway. SPT, serine palmitoyl transferase, SPHK, sphingosine kinase, SMase, sphingomyelinase

Sphingolipids (Figure 1 ) constitute a group of bioactive molecules, involved in numerous cellular processes. These lipids are engaged in cellular proliferation, differentiation, inflammatory responses, and apoptotic processes (Hannun & Obeid, 2002 ; Coant, Sakamoto, Mao, & Hannun, 2017 ). The central molecule and main second messenger of sphingomyelin signaling pathway is ceramide, an important lipid moiety that activates many kinases, phosphatases, and transcription factors (Hannun & Obeid, 2008 ). Ceramide can be generated in de novo pathway or by sphingomyelin degradation (Figure 1 ) (Hanada, 2003 ; Straczkowski & Kowalska, 2008 ). Among less important routes of ceramide synthesis are ceramide‐1‐phosphate conversion and so‐called *salvage pathway* (Hannun & Obeid, 2002 ). During the first step of ceramide de novo synthesis an enzyme serine palmitoyltransferase (SPT) catalyzes reaction of palmitoyl‐CoA and amino acid serine condensation. The product of this reaction is 3‐ketosphinganine, a molecule that is rapidly reduced to sphinganine (SFA). SFA undergoes acylation to dihydrocermiade. This molecule is converted into ceramide (by addition of trans 4,5‐double bond) or transferred to sphinganine‐1‐phophate (SFA1P) (Hanada, 2003 ). Another important source for ceramide generation is plasma membrane sphingomyelin (SM) hydrolysis. Activation of this process occurs in the presence of neutral (nSMase) or alkaline (alkSMase) isoforms of the enzyme: sphingomyelinase (Figure 1 ). Ceramide can be further metabolized in the presence of ceramidase (LASS4) to form sphingosine (SFO). In the last step of sphingomyelin signaling pathway SFO is phosphorylated into sphingosine‐1‐phosphate (Spiegel & Milstien, 2003 ). The last reaction is governed by the enzyme sphingosine kinase 1 (SPHK1) (Figure 1 ).

Previously published data concerning sphingolipid metabolism in the salivary glands are extremely scarce. Sugiya and Furuyama (1991 ), for instance, reported that sphingosine stimulates calcium mobilization in the rat parotid salivary glands. Furthermore, Suzuki, Sugiya, and Furuyama (2002 ) found that sphingosine inhibits signal transduction via inhibition of phospholipase C activity coupled to GTP‐binding protein. In one of the relatively recent studies Liu et al. (2010 ) proved that SPHK1 overexpression is associated with the salivary gland carcinoma and might be a novel predictive marker for adjuvant therapy. However, to the best of our knowledge, the activation of sphingomyelin signaling pathway in the salivary glands in the conditions of insulin resistance or diabetes has not been previously studied. Thus the aim of the present paper was to examine sphingolipid metabolism in the salivary glands of (pre)diabetic rats (exposed to high fat diet feeding or streptozotocin injection). For this purpose, we selected two major salivary glands, the parotid and submandibular one, that contribute up to 85% of unstimulated (basal) saliva flow (65% and 20% for the submandibular and parotid gland, respectively) (Humphrey & Williamson, 2001 ). Moreover, these organs represent two different types of metabolism (oxidative—PSG, glycolityc—SMSG) (Ibuki, Simões, & Nogueira, 2010 ). This last notion seems to be quite important given a different response pattern observed for some tissues, for example, skeletal muscles (Kurek et al., 2015 ) and the salivary glands (Matczuk, Zalewska, Lukaszuk, Garbowska, et al., 2016 ; Matczuk, Zalewska, Lukaszuk, Knas, et al., 2016 ), in the case of (pre)diabetic animals.

## 2 MATERIALS AND METHODS

### 2.1 Protocol of the experiment

The experiment was performed on male Wistar rats. All experimental procedures were approved beforehand by the Local Ethical Committee for Animal Experiments of Medical University of Bialystok. The animals were maintained in appropriate conditions with a stable temperature (21–22°C), humidity, and unrestricted access to food and water and on a reverse 12/12 hr light–dark cycle. The rats were next randomly allocated into one of the three groups, namely:

1. Control (C) group (*n* = 8) fed with standard rodent diet.
2. High Fat Diet (HFD) group (*n* = 8) fed for 5 weeks with a high caloric research diet containing 60% of energy derived from fats.
3. Streptozotocin induced diabetes (STZ) group (*n* = 8).

The rats (*n* = 24) entered the experiment at the age of 4 weeks. At that time eight of them were randomly allocated to high fat diet group, the remaining animals were maintained on a standard chow diet. In the 7th week part of the rats (*n* = 8) form standard rodent diet were randomly designated for streptozotocin injection. In this case streptozotocin (Sigma–Aldrich, St. Louis, MO) was given by intraperitoneal injection (dissolved in citrate buffer pH 5.4 at the dose of 60 mg/kg of body weight) in order to destroy pancreatic β‐cells. The injection was performed after an overnight fasting 14 days prior to the expected end of the experiment (final age of the rats: 9 weeks). Subsequent development of type 1 diabetes was confirmed by measuring fasting glucose level 72 hr after streptozotocin application. The development of insulin resistance was confirmed by measuring fasting plasma glucose and insulin level (with subsequent calculation of HOMA‐IR).

At the end of the experiment (after an overnight fasting) all rats were anesthetized by intraperitoneal injection of pentobarbital (80 mg/kg of body weight) and sacrificed. The parotid and submandibular salivary glands were excised, immediately frozen in aluminum clamps precooled with liquid nitrogen and stored in the temperature of −80°C until further analyses.

### 2.2 Salivary glands sphingomyelin content

Tissue samples were pulverized in aluminum mortar precooled with liquid nitrogen and transferred to tubes containing a solution of methanol and antioxidant (0.01% butylated hydroxytoluene). Lipid fractions were then isolated by the method described by Bligh and Dyer (1959 ). Sphingomyelin was extracted using thin‐lyer chromatography (TLC). The gel bands were scraped off from the plates after their reference to the distance traveled by standard (sphingomyelin, Sigma–Aldrich) and moved into tubes containing internal standard (pentadecanoic acid). After transmethylation the sphingomyelin fatty acids were analyzed using gas‐liquid chromatography (GLC). Hewlett–Packard 5890 Series II system equipped with a double flame‐ionization detector and Agilent CP‐Sil 88 capillary column was utilized. Total sphingomyelin concentration is shown as the sum of individual fatty acid species (expressed in nM/g of the tissue).

### 2.3 Salivary glands ceramide content

A small portion of the chlorophorm phase was transferred to a fresh tube containing C17‐sphingosine (Avanti Polar Lipids, UK) as an internal standard. The organic phase which contained ceramide was then hydrolyzed at the temperature of 90°C for 60 min in the solution of 1 M KOH in 90% methanol. The liberated sphingosine was subsequently analyzed using high performance liquid chromatography (HPLC). As a standard for the preparation of calibration curve n‐palmitoylsphingosine (Avanti Polar Lipids, UK) was used. The assessed amount of ceramide was adjusted with respect to the level of free sphingosine in the same sample.

### 2.4 Salivary glands sphinganine, sphinganine‐1‐phosphate, sphingosine, and sphingosine‐1‐phosphate contents

The quantities of the following lipids were measured according to the method described by Min, Yoo, Lee, Lee, and Lee (2002 ). In the first step of this procedure, prior to the samples homogenization and ultrasonication, internal standards (C17‐sphingosine and C17‐sphingosine‐1‐phosphate) (Avanti Polar Lipids, UK) were added. Sphingoid bases were converted to their o‐phthalaldehyde derivatives and then examined with HPLC system (ProStar, Varian, Inc.) equipped with fluorescence detector and C18 reversed‐phase column (Varian, Inc. OmniSpher 5, 4.6 × 150 mm).

### 2.5 Expressions of SPT‐1, SPHK1, nSMase, alkSMase, and LASS4 enzymatic proteins and Western blot

Western blotting procedures were applied to detect selected proteins (i.e. SPT‐1, SPHK1, nSMase, alkSMase, LASS4) of sphingolipid signaling pathways, as described in details elsewhere (Lukaszuk et al., 2015 ; Mikłosz et al., 2017 ). Briefly, the submandibular and parotid salivary glands were homogenized in ice‐cold RIPA buffer (50 mM Tris‐HCl, 150 M NaCl, 1 mM EDTA, 1% NP‐40, 0.25% Na‐deoxycholate, 1 mM phenylmethylsulfonyl fluoride, 1 μg/ml aprotinin, 1 μg/ml leupeptin, 1 μg/ml pepstatin, 1 mM sodium orthovanadate, 1 mM sodium fluoride). Protein concentration was then determined by means of BCA protein assay kit with bovine serum albumin as a standard. The samples were boiled at 95°C for 10 min in sample buffer containing 2‐mercaptoethanol. Next, proteins (40 μg of the total protein) were resolved using 10% SDS–PAGE and transferred to nitrocellulose membranes (0.75 A for 1 hr). The membranes were then blocked for 90 min at room temperature in TTBS buffer (50 mM Tris‐HCl, 130 mM NaCl, and 0.05% Tween‐20) containing 5% nonfat dry milk. Then the membranes were incubated overnight at 4°C with the corresponding antibodies at a dilution of 1:1000. The primary antibodies were purchased from Sigma–Aldrich (SPT1, SPHK1, LASS4) and (Santa Cruz Biotechnology, Dallas, TX) (nSMase, alkSMase, GAPDH). Thereafter, the membranes were incubated with anti‐rabbit or anti‐goat IgG horseradish peroxidase‐conjugated secondary antibody (1:3000, Santa Cruz Biotechnology). Immunoreactive protein bands were visualized using an enhanced chemiluminescence substrate (Thermo Scientific, Waltham, MA) and quantified densitometrically (Bio‐Rad Laboratories, Hercules, CA). Ponceau staining technique was performed to confirm equal protein loading in each lane on the blot membrane. The protein expression (Optical Density Arbitrary Units) was normalized to GAPDH expression. Finally, control group was set to 100 and the experimental groups were expressed relative to the control.

### 2.6 Statistical analysis

The obtained results are presented as mean and 1 standard deviation (SD). Statistical differences between the examined and control groups were assessed using one‐way ANOVA with a following post‐hoc test (pairwise Student's *t*‐test with Benjamini–Hochberg correction for multiple comparisons). If the test assumption were not met Kruskall–Wallis test with the succeeding post‐hoc pairwise Mann–Whitney U test (with Benjamini–Hochberg correction for multiple comparisons) were applied. Statistical significance level α was set at 0.05.

## 3 RESULTS

### 3.1 Sphingomyelin content

There were no significant differences in the SM contents between the C and HFD groups with respect to both the parotid and submandibular salivary glands (Tables 1 and 2 ). However, the rats from the STZ group in comparison with the C group were characterized by a significant increment (+22%) in SM content in the parotid salivary glands (*p* < 0.05, Table 1 ). Moreover, in the submandibular salivary glands a significant decrement (−15%) in SM concentration was noticed (C vs. STZ, *p* < 0.05, Table 2 ).

**Table 1:** Sphingomyelin [nmol/ml] and ceramide [pmol/ml] concentration in the parotid salivary glands

	Parotid salivary glands (PSG)		
	Ctrl (mean ± SD)	HFD (mean ± SD)	STZ (mean ± SD)
Sphingomyelin [nmol/ml]	1322.74 ± 155.465	1451.75 ± 169.350	1609.49 ± 89.092^a^
Ceramide [pmol/ml]	360.29 ± 60.079	294.37 ± 36.285	303.73 ± 64.64

^a^ Versus Ctrl (*p* < 0.05), *n* (per experimental group) = 8.

**Table 2:** Sphingomyelin [nmol/ml] and ceramide [pmol/ml] concentration in the submandibular salivary glands

	Submandibular salivary glands (SMSG)		
	Ctrl (mean ± SD)	HFD (mean ± SD)	STZ (mean ± SD)
Sphingomyelin [nmol/ml]	1680.78 ± 292.229	1692.23 ± 126.400	1434.23 ± 113.46^a^
Ceramide [pmol/ml]	128.16 ± 16.756	113.42 ± 9.190*	154.11 ± 14.125*

^a^ Versus Ctrl (*p* < 0.05), *n* (per experimental group) = 8.

### 3.2 Ceramide content

In the parotid salivary glands no significant differences in ceramide contents between any of the studied groups were noticed (Table 1 ). However, the rats from the HFD group were characterized by a significant reduction (−12%) in the ceramide content in the submandibular salivary glands (C vs. HFD, *p* < 0.05, Table 2 ). On the other hand, the STZ group was characterized by a significant increment (+20%) in the ceramide concentration in the submandibular salivary glands (C vs. STZ, *p* < 0.05, Table 2 ).

### 3.3 Sphingosine, sphinganine, sphingosine‐1‐phosphate, and spninganine‐1‐phosphate contents

**Figure 2:**
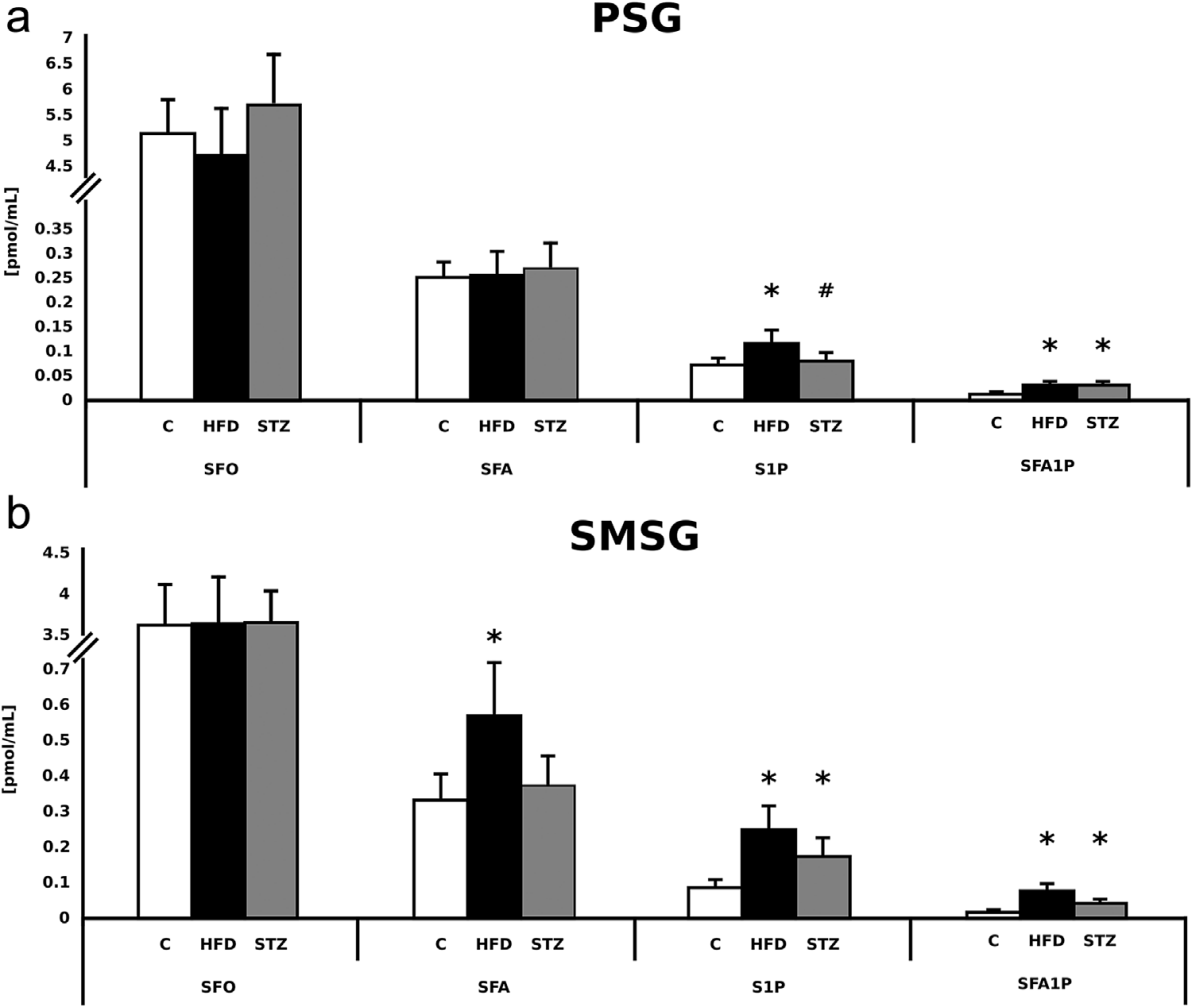
Efects of high fat diet feeding and streptozotocin injection on the salivary glands sphingolipid profile. (a) Parotid salivary glands. (b) Submandibular salivary glands. Group designations (*n* = 8 per group): C, control, HFD, high fat diet, STZ, streptozotocin. SFO, sphingosine, SFA, sphinganine, S1P, sphingosine‐1‐phosphate, SFA1P, sphinganine‐1‐phosphate. *Difference versus C (*p* < 0.05)

In both the parotid and submandibular salivary glands there were no significant changes in SFO content between the HFD and C group (Figure 2 a,b). Moreover, no significant changes in SFO contents were observed between the STZ and C group in both the parotid and submandibular salivary glands (Figure 2 a,b).

In the parotid salivary glands we noticed no significant differences in SFA concentration between any of the studied groups (Figure 2 a). However, the rats from the HFD group were characterized by a significant increment (+70%) in SFA content (C vs. HFD, *p* < 0.05, Figure 2 b) in the submandibular salivary glands. On the contrary, no differences between the STZ and C group with respect to SFA content in the submandibular salivary glands were noticed.

In the parotid salivary glands of rats from the HFD group in comparison with the C group there was a significant increment (+58%) in the amount of S1P (*p* < 0.05, Figure 2 a). With respect to the submandibular salivary glands we found a significant (+180%) increment in S1P (C vs. HFD, *p* < 0.05, Figure 2 b). Similarly, the rats from the STZ group were characterized by a significant increment in S1P concentration (+98%) in the submandibular salivary glands (C vs. STZ, *p* < 0.05, Figure 2 b).

The rats from the HFD group were characterized by a significant increment of SFA1P content (+138%) in the parotid salivary glands (C vs. HFD, *p* < 0.05, Figure 2 a). Moreover, the animals from the STZ group had significantly elevated SFA1P level (+136%) in the parotid salivary glands (C vs. STZ, *p* < 0.05, Figure 2 a). Furthermore, in the submandibular salivary glands from the HFD group we observed a substantial increment (+249%) in the amount of SFA1P (C vs. HFD, *p* < 0.05, Figure 2 b). Finally, the rats from the STZ group were characterized by a significant increment (+109%) in SFA1P concentration in the submandibular salivary glands (C vs. STZ, *p* < 0.05, Figure 2 b).

### 3.4 Expressions of SPT‐1, SPHK1, nSMase, alkSMase, and LASS4 enzymatic proteins

**Figure 3:**
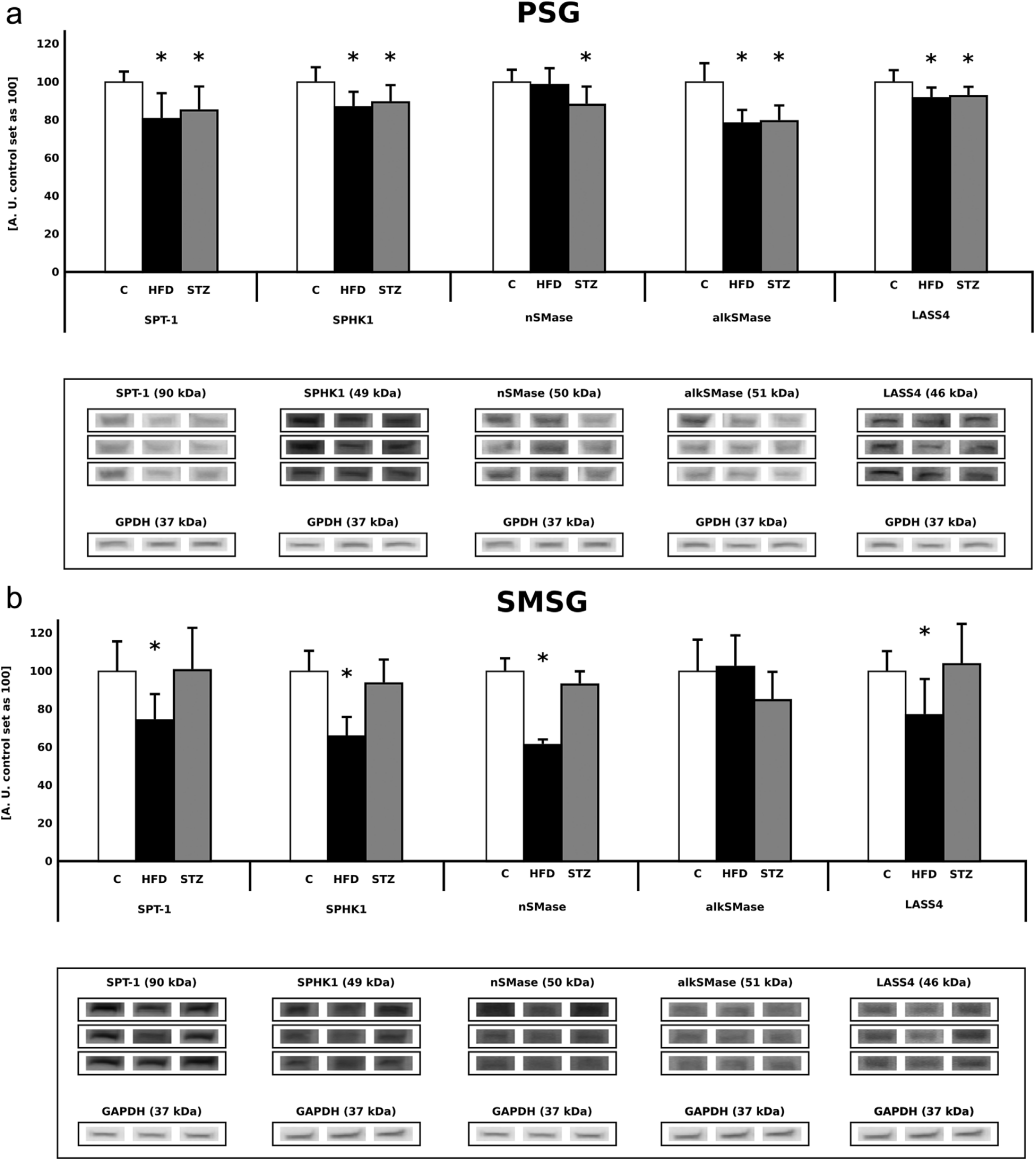
Efects of high fat diet feeding and streptozotocin injection on the salivary glands sphingolipid signaling pathway enzymes. (a) Parotid salivary glands. (b) Submandibular salivary glands. Group designations (*n* = 8 per group): C, control, HFD, high fat diet, STZ, streptozotocin. SPT‐1, serine palmitoyl transferase‐1, SPHK1, sphingosine kinase‐1, nSMase, neutral sphingomyelinase, alkSMase, alkaline sphingomyelinase, LASS4, ceramidase. *Difference versus C (*p* < 0.05)

In comparison with the C group, the rats from the HFD group were characterized by a significant decrement in the expression level of the following enzymes: SPT‐1 (−19%), SPHK1 (−13%), alkSMase (−21%), and LASS4 (−8%) in the parotid salivary glands (C vs. HFD, *p* < 0.05, Figure 3 a). However, no significant changes in nSMase expression in the parotid salivary glands were noticed between the HFD and C group (Figure 3 a). Moreover, the rats from the STZ group were characterized by a significant decrement in the amount of: SPT‐1 (−15%), SPHK1 (−10%), nSMase (−12%), alkSMase (−20%), and LASS4 (−7%, *p* > 0.05) in the parotid salivary glands (C vs. STZ, *p* < 0.05, Figure 3 a).

The rats from the HFD group were characterized by a significant decrement in the expression level of the following enzymes: SPT‐1 (−26%), SPHK1 (−34%), nSMase (−38%), and LASS4 (−23%) in the submandibular salivary glands (C vs. HFD, *p* < 0.05, Figure 3 b). However, no significant changes in the alkSMase expression in the parotid salivary glands were noticed (C vs. HFD, *p* > 0.05, Figure 3 b). Furthermore, we noticed no differences in the expression of the above‐mentioned enzymatic proteins in the STZ group in the case of submandibular salivary glands (C vs. STZ, *p* > 0.05, Figure 3 b).

## 4 DISCUSSION

In recent years much attention has been paid to the role of sphingolipids in the pathophysiology of many diseases (Knapp, Zendzian‐Piotrowska, Kurek, & Błachnio‐Zabielska, 2012 ). Although sphingolipid metabolism in the course of both diet induced insulin resistance and type 1 diabetes was studied in details in many peripheral tissues, including the liver (Yang et al., 2009 ), skeletal muscles (Ussher et al., 2010 ), and fat tissue (Russo, Ross, & Cowart, 2013 ), the activation of sphingolipid signaling pathway in the salivary glands has not been, so far, explored. This seems to be odd, since some of the previously published studies showed that the salivary glands of diabetic rats contain an increased amount of cytoplasm lipid droplets, thus additionally indicating a possible connection between their steatosis and the ongoing malfunction of these glands (Hand & Weiss, 1984 ; Anderson, Suleiman, & Garrett, 1994 ). Moreover, the salivary glands are a major agent engaged in the preservation of oral health and hygiene and patients with diabetes have significantly greater risk of contracting numerous buccal cavity maladies (e.g., periodontal disease, burning mouth syndrome, teeth decay, or mucosa ulcerations) (Carda et al., 2006 ; Leite et al., 2013 ; Negrato & Tarzia, 2010 ). Therefore, based on the before‐mentioned theoretical considerations and encouraged by our previous meaningful findings (Matczuk, Zalewska, Lukaszuk, Garbowska, et al., 2016 ; Matczuk, Zalewska, Lukaszuk, Knas, et al., 2016 ), we decided to explore the existing knowledge gap regarding the salivary glands sphingolipid metabolism in diabetic condition. The main cause of diabetes is either insufficient production of insulin by the pancreas or the failure of the body cells to properly respond to this hormone (Bonen, Chabowski, Luiken, & Glatz, 2007 ; Pociot & Lernmark, 2016 ). To replicate those conditions we employed high fat diet feeding (5 weeks, induction of peripheral tissue insulin resistance) (Harasim et al., 2015 ; Kurek et al., 2015 ) and streptozotocin injections (selective destruction of the pancreatic islets β‐cells) (Sakata, Yoshimatsu, Tsuchiya, Egawa, & Unno, 2012 ). The above‐mentioned treatments are commonly applied models of type 2 (pre)diabetic and type 1 diabetic state, respectively (Kurek et al., 2015 ; Kurek, Wiesiołek‐Kurek et al., 2014 ). For practical reasons we have narrowed our interests to two major salivary glands, namely the parotid and submandibular one. Together they contribute up to 85% of unstimulated (basal) saliva flow (65% and 20% for the submandibular and the parotid gland, respectively) (Humphrey & Williamson, 2001 ). Moreover, these organs represent two different types of metabolism (oxidative—PSG, glycolityc—SMSG) (Ibuki et al., 2010 ; Matczuk, Zalewska, Lukaszuk, Knas, et al., 2016 ). This last notion seems to be quite important given a different response pattern observed for some tissues (e.g., skeletal muscles (Kurek et al., 2015 ) and the salivary glands (Matczuk, Zalewska, Lukaszuk, Knas, et al., 2016 ) in the case of (pre)diabetic animals.

First of all, the most important finding of our study is an elevation in ceramide content observed in the submandibular salivary glands of type 1 diabetic rats (Table 2 ). Interestingly, nowadays an increasing amount of evidence supports the hypothesis that STZ induced Type 1 diabetes leads to an elevation of ceramide content (via the activation of its de novo synthesis) in many peripheral tissues, for example, in the liver and skeletal muscles (Blachnio‐Zabielska, Zabielski, Baranowski, & Gorski, 2010 ; Kurek, Wiesiołek‐Kurek et al., 2014 ). Similarly, in the present study, we noticed an increment in the SMSG ceramide content (Table 2 ) with concomitant elevation of SPT1 protein level (Figure 3 b), which indicates an increased activation of ceramide de novo synthesis. Interestingly, second main source for cellular ceramide generation, for example, sphingomyelin hydrolysis, was probably inhibited judging by the observed significant drop in the amount of SM in the submandibular salivary glands (−25% C vs. STZ, *p* < 0.05, Table 2 ). Although the expression of sphingomyelinases in the SMSG remained unchanged (C vs. STZ, Figure 3 b), an elevation in the ceramide content with concomitant reduction in the amount of SM strongly suggests increased hydrolysis of this lipid fraction (C vs. STZ, Table 2 ). Furthermore, in the discussed type of salivary glands (SMSG) in the rats with type 1 diabetes, ceramide is further metabolized to SFO and S1P (Figures 1 and 2b). This is revealed by the observed elevation in the submandibular salivary gland S1P content associated with the unchanged LASS4 level (C vs. STZ, *p* < 0.05, Figures 2 b and 3b). This observation is in line with previous reports concerning rat peripheral tissues characterized by high metabolic activity, including the liver and skeletal muscles (Blachnio‐Zabielska et al., 2010 ; Kurek et al., 2016 ). The obtained data strongly indicate that type 1 diabetes, which is a dynamic state with rapid onset and progression, leads to activation of sphingomyelin signaling pathway in the rats’ submandibular salivary glands.

On the other hand, our experiment demonstrated that obesity and type 2 prediabetic state cause different changes in sphingolipid metabolism in the submandibular salivary glands. First of all, we noticed that the rats fed with high fat diet were characterized by a decreased ceramide de novo synthesis (SMSG, C vs. HFD, *p* < 0.05, Table 2 ). This notion is supported by a decrement in ceramide concentration together with concomitant decrease of SPT‐1 protein level (submandibular salivary glands, C vs. HFD, *p* < 0.05, Table 2 , Figure 3 b). Moreover, it seems that in the SMSG of the HFD rats the hydrolysis of SM was also inhibited. This notion is indicated by the observed decrement in nSMase expression with no significant changes in SM concentration (C vs. HFD, *p* < 0.05, Table 1 , Figure 3 b). These results are in line with previously described observations concerning sphingolipids metabolism in diabetic rats livers and skeletal muscles (Kurek et al., 2015 ). Interestingly, we noticed an increment in the SMSG S1P content in the HFD group despite the decreased SPHK1 and LASS4 protein expressions (C vs. HFD, *p* < 0.05, Figures 2 b and 3b). However, it is important to emphasize that insulin resistance resulting from chronic high fat diet feeding does not activate sphingomyelin signaling pathway.

The second main type of the salivary glands, the parotid gland, differs from SMSG with respect to lipid metabolism which was previously described (Matczuk, Zalewska, Lukaszuk, Knas, et al., 2016 ). In our study the activation of ceramide de novo synthesis in the PSG seems to be inhibited in both the HFD and STZ group (Figure 3 a). This observation is confirmed by a significant decrement in the amount of SPT1 despite the lack of differences in ceramide concentrations (C vs. HFD and STZ, Table 1 , Figure 2 a). On the contrary, the results published so far regarding sphingolipids metabolism in other tissues, e.g. the liver and skeletal muscles, revealed an activation of ceramide de novo synthesis in the course of diet induced insulin resistance (Kurek et al., 2015 ; Kurek, Piotrowska, et al., 2014 ). This phenomenon strongly indicates that the PSG are organs with relatively low metabolic activity. Furthermore, an important and novel finding of our present research is the one regarding inhibition of SM hydrolysis in PSG. This suggestion is supported by a decrement in SMases levels observed in both experimental groups (PSG, C vs. HFD and STZ, Figure 3 a). The above‐mentioned data indicate that the activation of sphingomyelin signaling pathway in the PSG is inhibited in the course of both insulin resistance and type 1 diabetes and PSG is a type of salivary glands characterized by relatively low metabolic activity.

Moreover, SM concentration remained unchanged in the parotid glands of the insulin resistant animals and was even increased in the case of type 1 diabetic animals (Table 1 ). Finally, we noticed an increment in the content of S1P (C vs. STZ, *p *> 0.05, Figure 2 a) despite the fact that the expression levels of LASS4 and SPHK1 were decreased (C vs. STZ, *p* < 0.05, Figure 3 a). On the other hand, the insulin resistant rats from the high fat diet fed group were characterized by an increased S1P level in the PSG (Figure 2 a). The obtained data are in agreement with the previously published results concerning sphingolipid metabolism in other peripheral tissues (Fox et al., 2011 ; Kurek et al., 2015 ).

Closer scrutiny of the data presented in this paper seems to reveal an intriguing pattern regarding different submandibular and parotid salivary glands responsiveness. One may notice, for instance, an increased activation of sphingolipids signaling pathway in the submandibular salivary glands as compared to their parotid counterpart. The above is indicated by a higher magnitude of changes observed for several components of the pathway, for example, ceramide, sphinganine, or sphingosine‐1‐phosphate (Tables 1 –2, Figure 2 a,b). At first glance this seems to be rather a bit strange and counterintuitive. The parotid salivary glands are characterized by an oxidative, whereas the submandibular glands by a glycolytic, metabolism (Ibuki et al., 2010 ; Matczuk, Zalewska, Lukaszuk, Knas, et al., 2016 ). Greater aerobic metabolism is usually connected with grater reactive oxygen species (ROS) formation rate which in turn activates many pathological processes including inflammation and insulin resistance (Houstis, Rosen, & Lander, 2006 ; Martinez, 2006 ). If so, one would rather expect that the (pre)diabetic condition should activate sphingolipids signaling pathway more distinctively in the oxidative salivary gland (PSG), as it is evident in other tissues (e.g., oxidative vs. glycolytic skeletal muscle) (Kurek, Wiesiołek‐Kurek et al., 2014 ; Kurek et al., 2015 ). The explanation of that phenomenon lies, perhaps, in the recent study of Zalewska et al. (2015 ). In their research the authors compared antioxidant profile in the parotid and submandibular glands of type 1 diabetic rats in four different time periods (weeks: 2, 4, 10, 14). They found that the SMSG presented greater oxidational damage at the beginning of the experiment (Zalewska et al., 2015 ). This was probably a result of an increased ROS production that outmatched the tissue antioxidant defensive mechanisms (Zalewska et al., 2015 ). This could, at least partially, explain the inter‐glands difference (greater activation of sphingolipids signaling pathway in the SMSG) observed in our study, since oxidative stress is known to be associated with formation of ceramide and other sphingolipids (Martinez, 2006 ; Rochette, Zeller, Cottin, & Vergely, 2014 ).

**Figure 4:**
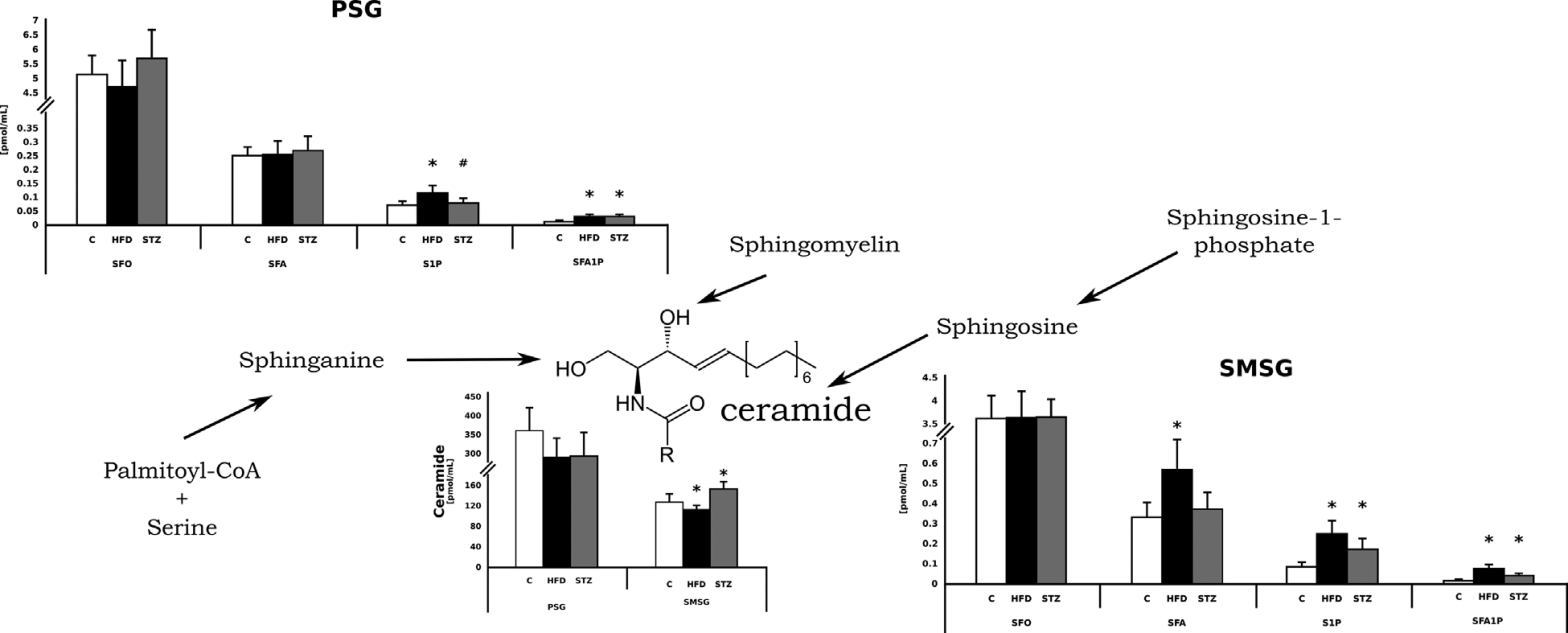
The effects of high fat diet and submandibular salivary glands on sphingolipid metabolism. C, control, HFD, high fat diet, PSG, parotid salivary glands, SMSG, submandibular salivary glands; STZ, streptozotocin. SFO, sphingosine; SFA, sphinganine; S1P, sphingosine‐1‐phosphate; SFA1P, sphinganine‐1‐phosphate. *Difference versus C (*p* < 0.05)

In summary, we explored and described, to the best of our knowledge for the first time, sphingolipid metabolism in the rats salivary glands in both diet induced insulin resistance and type 1 diabetic condition. Based on our results we conclude that the SMSG are characterized by somewhat higher metabolic activity (Figure 4 ). On the other hand, the changes in sphingolipids metabolism in the PSG were not expressed so clearly (Figure 4 ). Another interesting and novel finding of our study is an activation of sphingomyelin signaling pathway observed only in the course of type 1 diabetes, which is a metabolic condition with rapid onset and progression. On the contrary, long‐lasting insulin resistance, resulting from chronic high fat diet feeding lead to an inhibition of sphingomyelin signaling pathway in the rats salivary glands, which was manifested in an inhibition of ceramide de novo synthesis and accumulation of S1P (Figure 4 ). Although the results of our experiment delivered many interesting, novel and previously unpublished findings, in order to fully understand sphingolipids metabolism in diabetic rats salivary glands further studies are required.
